# Epigenetic understanding of gene-environment interactions in psychiatric disorders: a new concept of clinical genetics

**DOI:** 10.1186/1868-7083-4-1

**Published:** 2012-01-20

**Authors:** Takeo Kubota, Kunio Miyake, Takae Hirasawa

**Affiliations:** 1 Department of Epigenetics Medicine, Interdisciplinary Graduate School of Medicine and Engineering, University of Yamanashi, Yamanashi, 1110 Shimokato, Chuo, Yamanashi 409-3898, Japan

## Abstract

Epigenetics is a mechanism that regulates gene expression independently of the underlying DNA sequence, relying instead on the chemical modification of DNA and histone proteins. Although environmental and genetic factors were thought to be independently associated with disorders, several recent lines of evidence suggest that epigenetics bridges these two factors. Epigenetic gene regulation is essential for normal development, thus defects in epigenetics cause various rare congenital diseases. Because epigenetics is a reversible system that can be affected by various environmental factors, such as drugs, nutrition, and mental stress, the epigenetic disorders also include common diseases induced by environmental factors. In this review, we discuss the nature of epigenetic disorders, particularly psychiatric disorders, on the basis of recent findings: 1) susceptibility of the conditions to environmental factors, 2) treatment by taking advantage of their reversible nature, and 3) transgenerational inheritance of epigenetic changes, that is, acquired adaptive epigenetic changes that are passed on to offspring. These recently discovered aspects of epigenetics provide a new concept of clinical genetics.

## Background

Until recently, in clinical genetics, epigenetics was a minor field, of which two unusual genetic phenomena (genomic imprinting and X-chromosome inactivation (XCI)) were the main aspects under investigation. Based on the findings related to these phenomena, epigenetic disorders were considered to be very rare. However, as epigenetics has become more popular, it has developed into a huge research field that extends beyond genetics, encompassing not only biology and medicine, but also nutrition, education, health and social sciences. It now appears that epigenetics bridges the two major disease-causing factors (environmental and genetic) in medicine. Therefore, it is time to review epigenetics in the light of recent findings.

In this review, we explain the epigenetic mechanisms that cause congenital disorders, show examples of environmental factors that can alter the epigenetic status, and discuss recent topics in epigenetics, such as the possibility of its inheritance and the use of epigenetic strategies for the treatment of diseases.

### Epigenetics: a field that bridges genetic and environmental factors

It has long been thought that environmental and genetic factors are involved in the pathogenesis of common diseases such as cancer, diabetes, and psychiatric disorders [1-5]. For instance, environmental factors, such as drugs, viral infection, toxins and vaccines were proposed to be associated with the recent increase in the frequency of autism [6-9].

**Figure 1:**
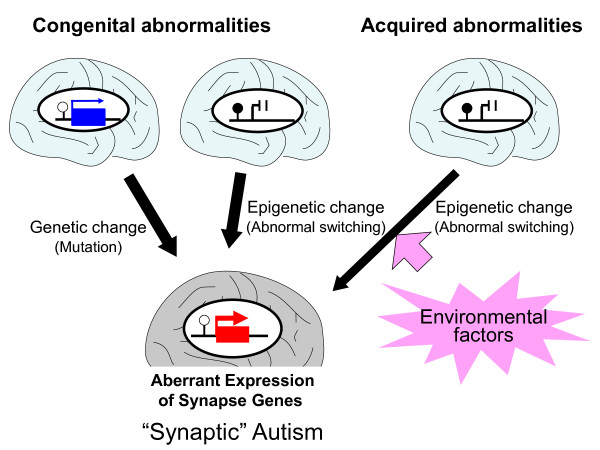
**Genetic and epigenetic understanding of autism**. Either *de novo *mutations in synaptic genes, congenital abnormalities of epigenetic control (for example, Rett syndrome), or acquired alterations of epigenetic control induced by various environmental factors can lead to synaptic dysfunction and resultin autism. Besides this category of 'synaptic autism', the categories of 'inflammatory autism' and 'splicing autism' are proposed [15].

In the meantime, a number of genes related to autism have been identified, which are mutated in a subset of autistic children. Most of these genes encode synaptic proteins, including synaptic scaffolding proteins, receptors, transporters, and cell-adhesion molecules [10,11]. A recent comprehensive study confirmed that there were differences between autistic and control brains in the expression levels of genes encoding synaptic proteins and proteins related to inflammation [12]. Based on these findings, autism is now considered as a 'synaptogenesis disorder' [13,14],, and designated 'synaptic autism' [15] (Figure 1, left).

It was recently reported that short-term mental stress caused by maternal separation during the neonatal period alters the epigenetic status of the glucocorticoid receptor (*Gr*) promoter in the rat hippocampus, which leads to changes in gene expression. This altered epigenetic status and abnormal gene expression persisted throughout life, and resulted in abnormal behavior [16]. This finding led us to posit a new paradigm in which epigenetics links genetics to environmental science [16]. Since then, similar observations have been reported [17,18], and epigenetics is now considered to be an intrinsic mechanism that bridges the gap between environmental and genetic factors (Figure 1, right).

### The first epigenetic phenomena to be associated with disorders

Genomic imprinting and XCI were the first two epigenetic phenomena discovered in mammals. Genomic imprinting is a unique genetic phenomenon in which only one of two parental alleles is expressed, while the other allele is suppressed. These genes are called 'imprinted genes'; the term 'imprinting' refers to a parent-of-origin specific epigenetic mark for suppression. Imprinting is considered to be a reversible mechanism, because the suppressed allele should be reactivated during gametogenesis when it is transmitted to next generation. For instance, the gene for small nuclear ribonucleoprotein polypeptide N (*SNRPN*) is only expressed by the paternal allele [19,20], but the maternally suppressed allele should be active during spermatogenesis when the allele is transmitted to the next generation via the male gamete. This phenomenon could not be interpreted by the usual genetic mechanisms, such as a change in the DNA sequence (that is, mutation), but can be explained by reversible epigenetic mechanisms based on chemical modifications, such as DNA methylation. In fact, differential DNA methylation was discovered in the promoter region of *SNRPN *between the paternal and maternal alleles [19].

XCI is another epigenetic phenomenon, which occurs only in females, because it compensates for the difference in the number of X chromosomes between females (XX) and males (XY), by silencing one of the X chromosomes in females [21].

Since the discovery of these two phenomena, abnormalities in these processes have been identified in a number of disorders, including Beckwith-Wiedemann syndrome (characterized by gigantism at birth [22]) Prader-Willi syndrome (characterized by obesity and features of obsessive-compulsive disorder), Angelman syndrome, (characterized by intractable epilepsy [19,20]), and XCI disorders such as ring Turner syndrome (which occurs when both the X and small ring X chromosomes are active, and is characterized by severe developmental delay that starts at birth [21]). Complete failure of XCI results in embryonic abortion [23,24]. These findings imply that proper epigenetic gene regulation is essential for normal development (Figure 1, middle).

### MeCP2: a molecule that bridges epigenetics and neuroscience

Two of the first disorders identified in an epigenetic molecule were ICF (immunodeficiency-centromeric instability-facial anomalies) syndrome [25] and Rett syndrome (RS). The latter is characterized by epilepsy, ataxia and autistic features [26,27]. Because it is an X-linked dominant disease (it is embryonic lethal in males, thus patients are all female), the X chromosome was analyzed to identify the causative gene. At first, it was thought that the gene encoded a synaptic protein. However, the identified gene, the methyl-CpG binding protein 2 (*MECP2*) gene, encodes a transcriptional repressor [26] that is rarely seen, thus this unexpected result introduced a new paradigm, 'epigenetics,' and highlighted the importance of epigenetics in the brain.

Once the gene was identified, the next step in RS research was to understand the pathogenesis of this disorder in relation to the function of MeCP2. Because MeCP2 is a transcriptional repressor, it was expected that the brains of patients with RS would have abnormal upregulation of neuronal genes[28], and in fact, several dysregulated neuronal genes have been identified [29-31]. Because RS has an autistic feature that is caused by epigenetic failure, it was speculated that autism can be caused not only by mutations of synaptic molecules (as described in the introduction) but also by the aberrant expression of these molecules; this has been confirmed, as a synaptic function has been proven for protocadherins, which depends on their targeting by MeCP2 [32].

The MeCP2 protein also stabilizes genomic DNA by suppressing L1 retrotransposition (a genetic phenomenon in which an L1 sequence is inserted into various genomic regions when the L1 sequence is hypomethylated) [33]. The DNA sequence is different in each neuron because L1 retrotransposition occurs somatically in neurons [34], and MeCP2 deficiency accelerates this retrotransposition, suggesting that there is greater variation in DNA sequences and in expression pattern in the brains of patients with RS than in the brains of controls [33], as retrotransposition-driven L1 insertions can affect expression of adjacent genes [35]. Therefore, although no differences have been found in the genome sequences of monozygotic twins with disease discordance for multiple sclerosis [36], some differences may be detected in the sequences of monozygotic twins with RS.

RS is a congenital disease, in which the neurological features do not start at birth, but are first detected in late infancy or childhood (1**-**3 years of age). This is because the patients are heterozygous females, thus on average, 50% of their cells are normal cells, in which the X chromosome carrying the normal allele expresses *MECP2 *under random XCI. In addition, MeCP2 does not encode a protein related to neurogenesis, but to neuronal maturation [37]. Therefore, it is possible that RS might be treatable if the level of MeCP2 could be supplemented to take it to the normal level during the maturation stage after birth. Indeed, this hypothesis was recently proven in the mouse model described below [38].

**Figure 2:**
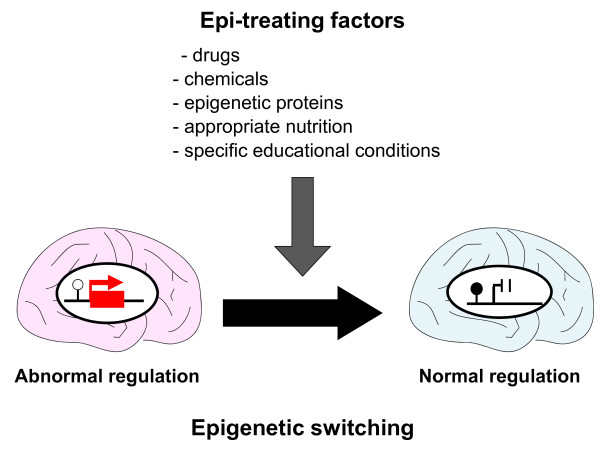
**Epigenetic switching with various epimutable factors**. A number of factors are known to exert epimutable effects. These factors alter the expression status by changing the epigenetic switches.

*Mecp2 *knockout mice mimic the neurological symptoms seen in patients with RS, including seizures, ataxic gait, and hind-limb clasping [39]. A new *Mecp2 *'knock-in' mouse model was created based on a first-generation *Mecp2*-knockout mouse, created by insertion of an 'exogenous' *Mecp2 *gene [38]. To produce this phenotype, the exogenous *Mecp2 *is initially silenced by an inserted stop codon, but it can be reactivated by treatment with tamoxifen (an estrogen analog), which causes the Cre-estrogen receptor fusion protein to translocate from the cytoplasm, where it is inactive, to the nucleus, where the Cre recombinase acts to recombine the two loxP sites that flank the inserted stop codon. Therefore, these mice exhibit neurological symptoms shortly after birth; however, after treatment with tamoxifen, the symptoms became much milder and the mice survived longer than the first-generation *Mecp2 *knockout mice. These results indicate that the developmental absence of MeCP2 does not irreversibly damage neurons and that the subsequent neurological defects are not irrevocable. Furthermore, the results indicate that neurodevelopmental disorders caused by mutations in epigenetic molecules or epigenetic gene dysregulation are potentially treatable after birth. However, this strategy cannot immediately be applied to humans, because it is not possible to generate a *MECP2 *knock-in human before birth. Thus, we need to identify chemicals that activate the expression of *MECP2 *in patients with RS (Figure 2).

In addition to experiments using chemicals as described above, recent experiments have shown that appropriate environmental conditions (for example, providing toys that stimulate the brain) could ameliorate the neurological features of *Mecp2 *knockout mice by altering gene expression and synaptogenesis in the brain [40-43]. These results suggest that it is important to provide a stimulating educational environment for patients with RS, as this can potentially alter the epigenetic status. Thus, epigenetics may provide useful scientific information for the assessment of specific educational conditions.

### Epigenetics: key to the genetic understanding of environmental factors

Epigenetic alterations are seen in various cancers, and are currently used clinically as diagnostic markers [44]. These alterations occur in response to internal or external environmental cues [45], and occur over a long time period during carcinogenesis. However, mental stress (for example, decreased pup licking and grooming, and arched-back nursing) induced in rat mothers had effects on their offspring, with alterations in the DNA methylation and histone acetylation status of the glucocorticoid receptor (*Gr*) promoter seen in the hippocampus of the pups during the first week of life [16]. This was the first indication that epigenetic changes can be induced by environmental stimuli over a short period. Since then, other environmental factors, including consumption of folic acid [46] and royal jelly (confirmed in honeybees, but not yet in mammals) [47], malnutrition during the fetal period [48], use of drugs for mental disorders [49-53], and neuronal stimulation [54], have been reported to alter the epigenetic status.

These factors do not affect the whole genome, but target specific genomic regions in certain tissues. Dietary protein restriction during pregnancy in rats results in DNA hypomethylation at the promoters of the *Gr *and peroxisomal proliferator-activated receptor alpha (*Pparα*) genes in the offspring's liver; folic acid supplementation prevented this hypomethylation even during the post-weaning period [46]. In a mouse model of depression induced by chronic social defeat stress [49], chronic administration of imipramine, a commonly used antidepressant, induced long-lasting histone H3 acetylation at the P3 and P4 promoters and H3-K4 dimethylation at the P3 promoter of the brain-derived neurotrophic factor (*Bdnf*) gene, with increased expression in the hippocampus. An antiepileptic drug, valproic acid (VPA), which is an inhibitor of histone deacetylases (HDACs), blocked seizure-induced aberrant neurogenesis by normalizing the expression of the HDAC-dependent glutamate receptor 2 gene (*GluR2*) in the rat hippocampus, which protected the animals from seizure-induced cognitive impairment [50]. In a study on mice in which the *Reln *promoter was hypermethylated by pretreatment with L-methionine, clozapine and sulpiride (atypical antipsychotics for schizophrenia and bipolar disorder) decreased DNA methylation at the reelin (*Reln*) promoter and the N-terminus of the 67 kDa glutamic acid decarboxylase (*Gad67*) promoter in the frontal cortex and striatum [51]. This demethylation effect of clozapine and sulpiride was enhanced in combination with VPA, and the effect was specific to the brain, as it was not observed in the liver [51]. This demethylation effect of VPA at the *Reln *and *Gad67 *promoters in the frontal cortex of mice was further confirmed by a different research group [52]; however, the precise mechanism underlying this demethylation process in the brain still remains to be elucidated. Lithium, another drug used to treat bipolar disorder, was found to have an epigenetic effect in a study on induced pluripotent stem cells (iPSCs). In this study, iPSC generation was enhanced with lithium treatment, which resulted in the downregulation of lysine-specific histone demethylase (LSD)1, an H3K4-specific histone demethylase, and a consequent increase in the endogenous expression of *Nanog*, an essential factor for induction of iPSCs [53].

These findings suggest that neurodevelopmental disorders such as autism can be caused not only by congenital genetic and epigenetic defects, but also by epigenetic dysregulation in the brain induced by various environmental factors (Figure 1, right). All of these findings were obtained through animal experiments, but there are also greater differences in epigenomic patterns between older monozygotic twins than between younger twins [55], suggesting that the epigenome is also affected by environmental factors in humans.

### Epigenetics: a concept for the transgenerational inheritance of acquired characteristics

It has long been believed that acquired characteristics are not inherited by the next generation, a belief based on Darwinian theory. However, Lamarck suggested that genetic changes can be influenced and directed by environmental factors, and DNA methylation is now thought to underlie this theory.

Epigenetic markers allow the transmission of gene activity states from one cell to its daughter cells; however, until recently, epigenetic marks were thought to be completely erased and then re-established in each generation. However, there have been several reports indicating that this erasure is incomplete at some loci in the genome of several model organisms, and that an epigenetic marker acquired in one generation can be inherited by the next generation. This phenomenon is now called 'transgenerational epigenetic inheritance' [56,57], and is an explanation of lamarckism, the idea of the heritability of acquired characteristics.

The transgenerational epigenetic inheritance of metastable epialleles was first demonstrated in a mouse strain, in which the methylation status of the *Axin (Fu) *allele, which is linked to the shape of the tail in the mature sperm, reflects the methylation status in the somatic tissue. In this strain, this allele did not undergo epigenetic reprogramming during gametogenesis [58]. This observation was recently confirmed in a different species, namely yeast, in which an aberrant epigenetic marker that was acquired in one generation after heat-shock treatment was inherited by the next generation [59]. Furthermore, it was recently reported that mental stress (separation from the mother) not only changes the DNA methylation status in the brain of separated pups, but also changes it in the sperm of the males, and the changed status is transmitted to the next generation. In the next generation, the changed status is visible in the brain of offspring, and also produces alterations in the corticotropin releasing factor receptor 2 (*Crfr2*) gene expression and in the animal's behavior [60,61]. Although further evidence is needed, these findings imply that a susceptibility to mental disorders that is inherited by succeeding generations depends not only upon specific genomic changes (mutations in genes) but also upon specific epigenomic changes that are initially induced by environmental factors. Future studies are necessary to identify therapeutic strategies that take advantage of the reversibility of stress-induced epigenetic modifications. These studies could also help us to identify appropriate environments for maintaining a healthy physical and mental condition [62,63].

### Future perspectives

The clinical application of epigenomic information has improved in recent years. Its first application was a single gene-based DNA methylation assay to diagnose two imprinted disorders (Prader-Willi and Angelman syndromes) by taking advantage of the differential methylation present in a CpG region within an imprinted gene [20]. Recently, a microarray-based epigenomic assay has been developed as a second-generation test, which covers CpG sites in an entire region of a single chromosome [64]. More recently, a high-density BeadChip-based epigenomic assay has been developed as a third-generation test, and now covers 450,000 CpG sites distributed throughout the human genome. Using this method, a methylated site that was specific to heavy smokers was discovered within a gene that is possibly associated with cardiovascular complications [65].

Another important application of epigenetics relates to folic acid, which is a nutrient that provides methyl residues and is essential for the maintenance of DNA methylation. Folic acid is used to prevent neural tube defects such as spina bifida, and it is known that folic acid supplements can alter DNA methylation status [66,67]. Folic acid supplements can have a positive effect on several features of autism in children, although the underlying mechanism is not completely understood [68-70]. Folic acid is expected to exert a global effect on the genome; however, if we can identify genes in which the epigenome is changed in particular disorders (for example, the *SNRPN *gene in Prader-Willi syndrome during gametogenesis [19,20] and the coagulation factor II (thrombin) receptor-like 3 (*F2RL3*) gene in heavy smokers [65]), it might be possible to selectively restore the specific epigenomic status of the causative gene region. One method is to use pyrrole-imidazole (PI) polyamides, which are small chemicals that recognize and attach to the minor groove of DNA, and can be designed to target any DNA sequences. PI polyamide can be attached to inhibitors of DNA methylation or histone deacetylases [71], and it was recently reported that such a construct was delivered to a target gene and altered its expression [72].

As discussed above, acquired characteristics can be inherited by the next generation as an epigenetic marker, as suggested by Lamarck. Recent research on animals has shown that behavioral characteristics can be inherited [60]. Thus, if gene-specific epigenomic therapy using PI polyamides could be delivered to the affected genes (for example,, *Crfr2 *[61]), it might correct the altered epigenomic status, gene expression and behavior of the subject, and thus might prevent inheritance of the abnormal epigenetic status by future generations.

Recent sequencing technology has led to a precise understanding of the sequence structure of the human genome, and revealed the presence of copy-number variations (CNVs), which are associated with susceptibility to common diseases [73,74]. The presence of CNVs is currently a more favored genetic concept than epigenetics in some psychiatric disorders, such as autism [75]. However, the advantage of studying epigenetics over CNVs is that if we can understand the epigenetic basis of the inheritance of acquired characteristics, it might be possible to develop a new therapeutic strategy using the intrinsic reversibility of epigenetics and also to develop a new method of prevention can be developed for the following generation. Therefore, further understanding of interactions between genes and environment with respect to epigenetics is important, and will provide a new concept of clinical genetics.

## Conclusions

The failure of epigenetic gene regulation is known to cause various rare congenital disorders. However, this dysregulation also causes common diseases that are induced by environmental factors, as the epigenetic status is affected and changed by various environmental factors. Furthermore, the changed epigenetic status in the genome can be transmitted to the succeeding generations. Therefore, a precise understanding of the interactions between genes and environment in the light of epigenetics is necessary, and will form a new concept of clinical genetics.

## List of abbreviations

CNVs: copy-number variations; Crfr2: corticotropin releasing factor receptor 2; F2RL3: coagulation factor II (thrombin) receptor-like 3; MECP2: methyl-CpG binding protein 2; SNRPN: small nuclear ribonucleoprotein polypeptide N; XCI: X-chromosome inactivation.

## Competing interests

None of the authors has any competing interests associated with the studies described in this review article.

## Authors' contributions

TK drafted the manuscript. KM participated in writing the section entitled 'MeCP2: a molecule that bridges epigenetics and neuroscience'. TH participated in making the figures and helped to draft the manuscript. All authors read and approved the final manuscript.
